# Psychological Impact of AI-Simplified Brain MRI Reports: A Randomized Trial of Patient Understanding, Anxiety, and Health Literacy

**DOI:** 10.3390/jcm15114158

**Published:** 2026-05-28

**Authors:** Mohammad Alarifi, Jake Luo, Abdulrahman Jabour, Yazeed Alashban, Haitham Alahmad, Alhanouf Alshedi, Mansour Almanaa

**Affiliations:** 1 Radiological Sciences Department, College of Applied Medical Sciences, King Saud University, Riyadh 11451, Saudi Arabia; mohalarifi@ksu.edu.sa (M.A.); yalashban@ksu.edu.sa (Y.A.); hnalahmad@ksu.edu.sa (H.A.); aalshedi@ksu.edu.sa (A.A.); 2 College of Health Sciences, University of Wisconsin–Milwaukee, Milwaukee, WI 53211, USA; jakeluo@uwm.edu; 3 Health Informatics Department, Faculty of Public Health and Tropical Medicine, Jazan University, Jazan 45142, Saudi Arabia; ajabour@jazanu.edu.sa

**Keywords:** artificial intelligence, radiology reporting, patient-centered care, health literacy, anxiety

## Abstract

**Background/Objectives**: Immediate patient access to radiology reports has increased the need for communication that patients can understand, yet it remains unclear whether simplifying report language improves comprehension without worsening psychological distress. This study aimed to determine whether AI-based simplification of a brain MRI report improves patient understanding, to assess whether anxiety differs between standard and AI-simplified reports, and to examine the relationships among anxiety, report understanding, and health literacy. **Methods**: We conducted a minimal-risk, survey-based randomized experimental study using Qualtrics and Amazon Mechanical Turk. A total of 803 participants were randomized 1:1 to view either an original radiology report (control, *n* = 402) or an AI-simplified version of the same report (intervention, *n* = 401). The stimulus was a single de-identified brain MRI/MRV report. Primary outcomes were report understanding and post-exposure anxiety, and secondary measures included radiology literacy and general health literacy assessed with the Short Test of Functional Health Literacy in Adults (S-TOFHLA). Between-group differences were analyzed using Mann–Whitney U tests, and associations between variables were examined using correlation analyses. **Results**: Participants who received the AI-simplified report achieved significantly higher understanding scores than those who viewed the original report (mean 5.78 ± 1.31 vs. 5.61 ± 1.49; *p* = 0.007). Anxiety scores were similar between groups (mean 3.24 ± 0.84 vs. 3.23 ± 0.85; *p* = 0.103). A positive correlation was observed between anxiety and general health literacy (r = 0.283, *p* < 0.001), and report understanding was also positively correlated with anxiety (r = 0.182, *p* < 0.001). Age was negatively associated with anxiety, whereas income showed a weak positive association. **Conclusions**: AI-based simplification improved patient understanding of radiology reports but did not reduce anxiety. Greater understanding was associated with higher anxiety, suggesting that clearer language alone may be insufficient to address the emotional burden of reading radiology results without clinical context or reassurance.

## 1. Introduction

The implementation of the 21st Century Cures Act has fundamentally shifted the paradigm of radiology reporting by mandating immediate patient access to electronic health information [1]. This policy change has accelerated patient access to imaging results through online portals, often allowing patients to view radiology reports before a clinician has had the opportunity to explain the findings or provide reassurance. While this approach supports transparency, autonomy, and patient engagement, it also creates new communication challenges when complex or uncertain medical information is delivered without clinical context. The article “Communication Skills Training for Oncology Clinicians After the 21st Century Cures Act: The Need to Contextualize Patient Portal–Delivered Test Results” emphasizes that direct release of results can shorten waiting time, but ambiguity in portal-delivered results may also increase anxiety and lead patients to seek clarification through clinicians, internet searches, family members, or other sources [2].

While this transparency is intended to empower patients, it has exposed a critical disconnect: radiology reports are technically complex documents drafted for referring physicians, not laypeople [3,4]. Consequently, patients frequently struggle to interpret their findings, leading to confusion, distress, and an increased workload for ordering providers who must translate these technical documents [5]. Recent evidence confirms that traditional radiology reports may be difficult for patients to process because they contain specialized terminology, probabilistic language, and diagnostic expressions that may be misinterpreted by non-clinicians. The systematic review “The impact of different radiology report formats on patient information processing” reported that radiology reports are primarily written for health professionals and that patients may experience difficulty understanding jargon and terminology, which can contribute to anxiety and confusion [6]. This is particularly important because poor understanding of radiology results may increase unnecessary physician consultations, follow-up testing, emergency visits, and patient worry [6].

Health literacy and radiology-specific literacy are central to this problem. Even when patients can access their reports, they may not have the medical background needed to distinguish clinically serious findings from incidental or low-risk findings. The article “Patient experiences and anxiety related to medical imaging: challenges and potential solutions” highlights that patient preparation for medical imaging should include risk communication, procedural information, sensory information, behavioral instruction, and psychosocial support. It also notes that health literacy remains low and that interventions are needed to improve patient understanding and outcomes related to health literacy [7]. Therefore, improving readability alone may not be sufficient unless patients are also supported in understanding the meaning, urgency, and next steps related to the report.

Historically, the “patient-friendly” report has been difficult to scale, often relying on manual creation or static definitions [8]. However, the emergence of Large Language Models (LLMs) such as ChatGPT (OpenAI, San Francisco, CA, USA) has provided a scalable mechanism to translate medical jargon into plain language [9,10,11]. Recent studies suggest that LLMs can simplify reports to an appropriate reading level [12,13] and potentially improve comprehension [14]. This aligns with growing evidence that modified report formats, such as lay summaries, glossaries, patient-oriented definitions, and anatomic illustrations can improve patients’ satisfaction, perceived usefulness, and understanding of radiology reports. A systematic review concluded that modifying traditional radiology reports with lay language, glossaries, or illustrations enhances patient information processing and may reduce unnecessary insecurity, confusion, anxiety, and physician consultations [6]. At the same time, the same review cautioned that the role of AI and LLMs in presenting radiology information requires careful regulation because AI-generated explanations may be inaccurate if the input is incomplete, unchecked, or misunderstood [6].

Despite these technological advancements, radiologists retain valid concerns regarding the direct release of reports. A primary concern is that unguided access to complex medical information induces “scanxiety,” [15] a phenomenon where patients experience intense distress while waiting for or interpreting results [16]. The emotional burden of imaging is not limited to the scan itself; it may occur before the examination, during the waiting period, when results are released, and when patients attempt to interpret the report. One study describes scanxiety as an anticipatory emotional response involving fear, distress, and hypervigilance when awaiting or viewing imaging results. It also emphasizes that serious or uncertain findings delivered without context may compromise patient wellbeing [15]. Similarly, another study notes that anxiety and distress are common but often insufficiently managed in radiology and radiotherapy, and that imaging procedures such as MRI, CT, and PET can heighten distress through uncertainty, confinement, anticipation, and fear of results [17].

While it is often assumed that simplifying reports will reduce this anxiety by removing uncertainty, this relationship remains under-explored in randomized settings. Furthermore, prior research has largely focused on the accuracy of AI outputs [18] rather than the psychological impact on the patient. The prevailing hypothesis among radiologists has been that this anxiety is driven by difficult terminology; in other words, patients become anxious because they are confused by the language of the report [19]. A recent systematic review published in the Journal of the American College of Radiology traces this apprehension over decades, illustrating that radiologists have consistently cited the “medical terminology” barrier and the consequent patient anxiety as a primary reason to withhold or delay reports [20]. This review highlights a longstanding consensus that specialized terminology serves as an anxiety-provoking “black box” for laypeople, necessitating clinician intermediation to decode findings before patients view them [19,20]. Furthermore, studies analyzing patient forum discussions confirm that patients themselves frequently attribute their distress to their inability to decipher technical jargon, reinforcing the clinical community’s assumption that confusion is the root cause of their fear [21]. However, recent work on patient portal communication suggests a more nuanced problem: patients may desire rapid access to results while still needing guidance, interpretation, and reassurance to understand what the findings mean for their own clinical situation. One study specifically argues that clinicians now need skills to frame and clarify portal-delivered results because patients may encounter technical, confusing, or upsetting language before a direct clinical discussion occurs [2].

This study aimed to address four key objectives: (1) to determine whether AI-based simplification improves patient understanding of radiology reports, even among individuals with relatively high baseline comprehension; (2) to examine the relationship between anxiety and health literacy in the context of radiology report interpretation; (3) to assess whether anxiety levels differ between participants who received the standard radiology report and those who received the AI-simplified report; and (4) to investigate the association between report understanding and anxiety, including whether greater understanding is associated with lower anxiety.

## 2. Materials and Methods

### 2.1. Study Design

**Figure 1:**
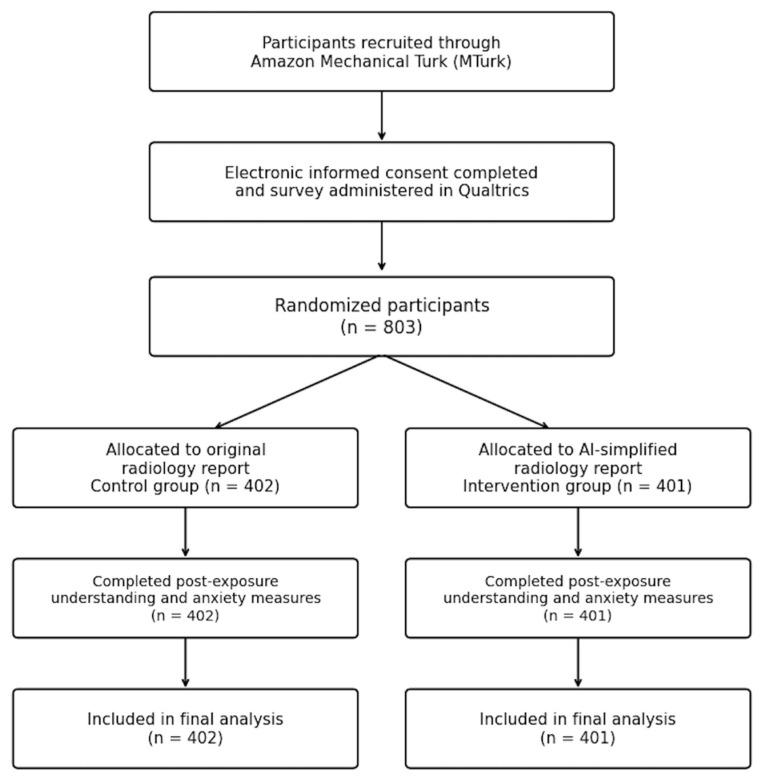
Participant flow diagram showing recruitment, consent, randomization, group allocation, completion of post-exposure measures, and final analysis.

This study was a minimal-risk, survey-based randomized experimental study in which participants were exposed to either an original radiology report or an AI-simplified version of the same report. No clinical intervention, diagnosis, treatment, or patient-care decision was performed as part of the study. Participants were randomly assigned in a 1:1 ratio using a computer-generated randomization procedure to either (1) an intervention arm that received the AI-simplified report or (2) a control arm that received the original radiology report only. The study was reported in accordance with relevant CONSORT principles for randomized trials, and a participant flow diagram was added to improve transparency of recruitment, randomization, allocation, and analysis (see Figure 1).

### 2.2. Study Material and AI Prompt Development

A single de-identified radiology report describing a male brain MRI with MR venography (MRV) was used as the standardized stimulus across study arms. To operationalize a realistic “patient-like” request for simplification, we used a two-step approach with ChatGPT 5.2 Thinking. First, we asked ChatGPT to generate common questions patients typically ask when seeking clarification of radiology reports. This step was intended to avoid using an investigator-created prompt that may not reflect how patients naturally ask for help when they encounter unfamiliar terminology in a radiology report. Instead, the goal was to identify patient-centered wording that resembled real-world information-seeking behavior. Second, we asked ChatGPT and Gemini (Google, Mountain View, CA, USA) to rank the candidate questions (1 = most frequent) based on the types of radiology-report clarification requests it most commonly receives from users; both ranked “Can you explain this radiology report in simple terms?” as the most frequent, and we therefore adopted it as the standardized simplification prompt (see Table 1). The ranking process was used only to support prompt selection and was not treated as empirical evidence of patient behavior. Rather, it served as a practical prompt-development step to select the question most likely to represent a broad, general request for report simplification.

Table 1 summarizes the candidate patient-like questions and the rankings provided by ChatGPT and Gemini. Lower numbers indicate questions judged to be more commonly asked, with rank 1 representing the most frequent type of clarification request. The rankings show that both models independently placed the general simplification question, “Can you explain this radiology report in simple terms?”, as the most frequent request. This agreement supported the use of this wording as the final intervention prompt because it was broad, understandable, and applicable to the entire report rather than being limited to a single term, finding, or report section.

This prompt was chosen because it aligns with documented patient information needs in radiology communication. In a prior online discussion forum analysis (659 patient questions), the most common category of questions concerned the radiology report (35.50%), and within that category the leading subtheme was patient understanding of the report (26.49%) [21]. MRI reports were also a frequent source of concern. Accordingly, selecting a plain-language explanation prompt reflects the dominant, real-world patient intent: to understand the meaning of radiology report content [21]. Using this patient-centered intent, the intervention report was generated using a large language model instructed to simplify the original report by producing a plain-language explanation while preserving the clinical meaning of the findings.

### 2.3. Participants, Recruitment, Randomization, and Study Workflow

Participants were recruited online through an Amazon Mechanical Turk (MTurk) Human Intelligence Task (HIT) (Amazon, Seattle, WA, USA), and the survey was administered using the Qualtrics platform (Qualtrics, Provo, UT, USA). Eligible participants who completed the survey received US $0.25 as compensation. A total of 803 participants completed the study and were included in the analysis: 401 in the intervention group and 402 in the control group. The final sample size was considered adequate for this online randomized survey because the target accessible population exceeded 10,000 individuals. Using a conservative large-population sample size approach with a 95% confidence level, 5% margin of error, and maximum variability assumption (*p* = 0.50), the minimum required sample is approximately 384 participants. Therefore, the final sample of 803 participants exceeded this threshold and provided balanced allocation across the two study arms, with more than 400 participants in each group. Randomization was implemented automatically by the Qualtrics system using a computer-based random allocation procedure to ensure that each participant had an equal probability of assignment to either arm. Allocation occurred immediately after consent and prior to report exposure. The intervention group viewed the AI-simplified report, while the control group viewed the original report only (provided in the Supplementary Materials).

After consenting and being randomized, participants completed the following sequence:

- Exposure to the assigned report (original vs. AI-simplified);
- Assessment of understanding and anxiety immediately following exposure;
- Completion of health literacy and radiology literacy measures and additional survey variables.

### 2.4. Outcome Measures and Literacy Assessment

The primary outcomes of this study included patient comprehension and psychological response following exposure to radiology reports. Understanding of the radiology report was assessed using a post-exposure comprehension quiz specifically tailored to the content of the presented report. Scores were calculated by summing correct responses, with higher totals indicating a superior level of comprehension. Simultaneously, psychological response (report-related worry/anxiety and pain-focused rumination) was evaluated immediately after report exposure using three brief Likert-type items embedded in the survey (see Supplementary Materials). These items were adapted from established validated measures/item banks (health worry from the Health Anxiety Inventory/Short Health Anxiety Inventory; unease/restlessness from PROMIS Anxiety; and pain-related rumination from the Pain Catastrophizing Scale), with wording modified to anchor responses specifically to reading the radiology report. Responses ranged from not at all to extremely, and item scores were analyzed as continuous outcomes, with higher values reflecting greater distress [22,23,24].

The study also incorporated secondary measures to account for baseline literacy levels that might influence report interpretation. Radiology literacy was quantified using the Radiology Literacy Tool (RLT), an instrument developed to evaluate patient familiarity with specialized radiological terminology and concepts [25]. General health literacy was measured via the Short Test of Functional Health Literacy in Adults (S-TOFHLA). This 36-item reading comprehension assessment (score range 0–36) allowed for the categorization of participants into three distinct literacy levels: inadequate (0–16), marginal (17–22), and adequate (23–36) [26].

### 2.5. Statistical Analysis

All statistical procedures were performed using IBM SPSS Statistics (Version 23) (IBM Corp., Armonk, NY, USA). The analysis plan was structured to provide a descriptive characterization of the study population, followed by inferential tests to evaluate between-group differences and variable associations.

To address the study’s primary and secondary outcomes, several comparative and correlational tests were employed:

- Between-Group Comparisons: Differences in report understanding scores, anxiety levels, radiology literacy, and health literacy were compared between the experimental groups. Due to the non-parametric nature of the data distribution, Mann–Whitney U tests were utilized for these comparisons.
- Categorical Analysis: Health literacy scores were stratified into inadequate, marginal, and adequate categories. The distributions of these categories across groups were examined using descriptive statistics.
- Association Testing: To explore the relationships between demographic factors (age, income) and outcome variables (health literacy, radiology literacy, report understanding, and total anxiety), Spearman’s rho or Pearson’s r correlation coefficients were calculated, with the selection based on the scale and distribution of each specific variable.

### 2.6. Ethical Approval

The study protocol was reviewed and granted Exempt Status (Category 2) by the University of Wisconsin–Milwaukee Institutional Review Board (IRB #: 20.230) in accordance with 45 CFR 46.104(d), confirming that the study posed minimal risk to participants. Ethical oversight was important because participants were exposed to radiology-report content that could potentially generate worry, uncertainty, or emotional discomfort, even though the report was fully de-identified and presented in a hypothetical research context. Before participation, all individuals reviewed an electronic informed consent statement describing the study purpose, voluntary nature of participation, survey procedures, potential risks, and confidentiality protections. Participants were informed that they could discontinue the survey at any time without penalty. Recruitment was conducted through Amazon Mechanical Turk (MTurk), and data were collected through Qualtrics. No identifiable health information was collected, responses were analyzed only in aggregate form, and no attempt was made to identify individual participants. These procedures were used to protect participant privacy, maintain anonymity, and support ethical assessment of both report understanding and anxiety.

## 3. Results

A total of 803 participants completed the study and were included in the primary analyses. The study population was highly educated, with 69.4% (*n* = 557) holding a bachelor’s degree and 12.2% (*n* = 98) holding a master’s or doctoral degree (see Table 2). Consistent with this educational background, baseline radiology literacy scores were high across the entire sample, with a mean of 8.20 out of 10 (see Table 2). Demographic characteristics, health literacy levels, and radiology literacy scores were well-balanced between the control and intervention groups (*p* > 0.05 for all comparisons), confirming successful randomization (see Table 3).

Table 3 presents the main study outcomes by randomized group, including report understanding, radiology literacy, general health literacy, and anxiety. This table was used to evaluate whether the AI-simplified report improved comprehension while also examining whether the two groups remained comparable in baseline literacy-related measures.

**Figure 2:**
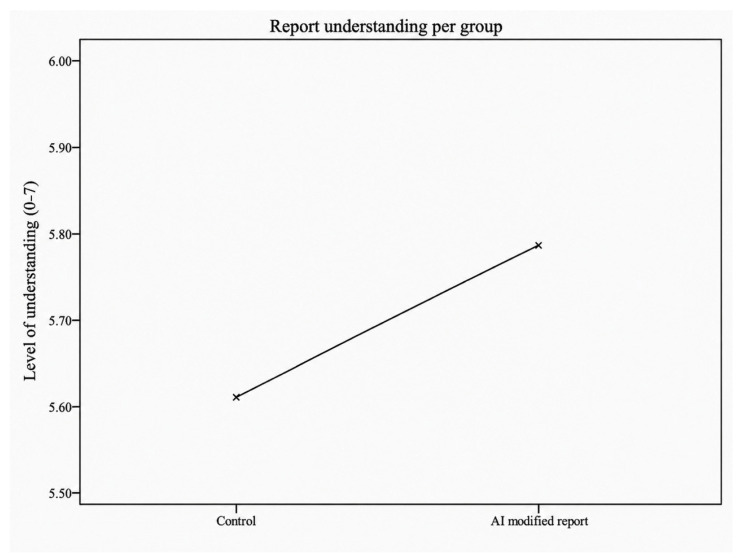
Mean report understanding scores by randomized group. Participants who received the AI-simplified report had a higher mean understanding score than those who received the original report.

**Figure 3:**
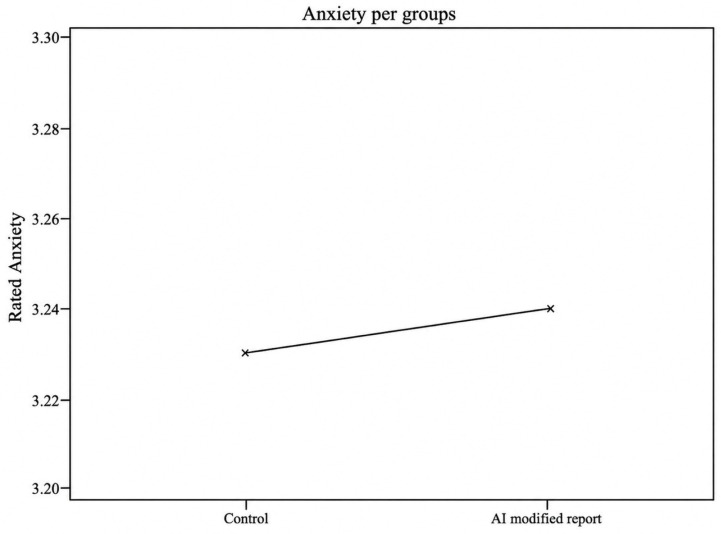
Mean anxiety scores by randomized group. Mean anxiety scores were similar between the original report and AI-simplified report groups, indicating that report simplification did not reduce anxiety.

**Figure 4:**
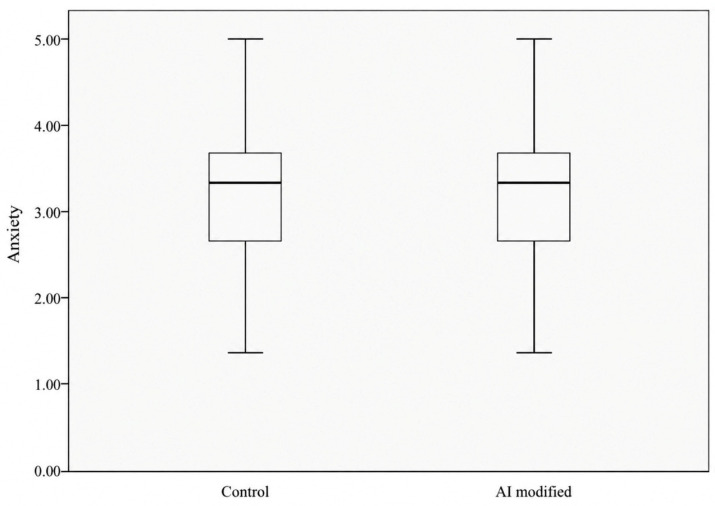
Distribution of anxiety scores by randomized group. The boxplot shows similar anxiety distributions across the two groups, supporting the finding that AI simplification did not independently lower report-related anxiety.

Figure 2 shows the difference in mean report understanding scores between the original report and AI-simplified report groups. Figure 3 shows the mean anxiety scores by group, and Figure 4 presents the distribution of anxiety scores across both groups. Together, these figures visually support the main finding that AI simplification improved understanding but did not meaningfully reduce anxiety. The standardized effect size for the difference in report understanding was small (Cohen’s d ≈ 0.12), indicating that although the difference was statistically significant, its practical magnitude was limited. The between-group difference in anxiety was negligible (Cohen’s d ≈ 0.01).

Regarding the primary outcome of comprehension, AI-based simplification produced a statistically significant improvement in patient understanding. Participants randomized to the AI-simplified report achieved higher Report Understanding Scores (mean 5.78 ± 1.31 out of 7) compared with those who viewed the original radiologist-authored report (mean 5.61 ± 1.49; *p* = 0.007) (see Table 3 and Figure 2). This difference indicates that AI simplification confers incremental comprehension benefits even within a cohort that possesses high baseline literacy.

In terms of psychological impact, however, no significant difference in anxiety levels was observed between the study arms. As shown in Figure 3, the mean anxiety scores were nearly identical between the AI-modified group and the control group, with a mean score of 3.24 ± 0.84 in the AI-modified group compared with 3.23 ± 0.85 in the control group (*p* = 0.103). This indicates that although AI simplification improved report understanding, it did not produce a measurable reduction in average anxiety. Figure 4 further supports this finding by showing a similar distribution of anxiety scores across both groups. The comparable spread and overlap between groups suggest that the simplified report did not substantially shift anxiety responses at the participant level. Together, Figure 3 and Figure 4 demonstrate that the psychological response to radiology-report information was not meaningfully changed by simplification alone.

Correlation analyses revealed distinct associations between anxiety, literacy, and comprehension. When classified by health literacy levels, 9.3% of participants had inadequate literacy, 63.4% had marginal literacy, and 27.3% had adequate literacy (see Table 4). A statistically significant positive correlation was observed between total anxiety and general health literacy (r = 0.283, *p* < 0.001), indicating that participants with higher health literacy reported higher levels of anxiety. Similarly, a statistically significant positive correlation was found between Report Understanding Scores and anxiety (r = 0.182, *p* < 0.001). Demographic factors also influenced anxiety levels; a negative correlation was observed between age and anxiety (ρ = −0.122, *p* = 0.001), while income showed a weak positive correlation with anxiety (ρ = 0.072, *p* = 0.041).

## 4. Discussion

### 4.1. The Efficacy of AI in Bridging the Comprehension Gap

This study demonstrates that AI-based simplification of radiology reports leads to a statistically significant improvement in patient understanding compared with standard radiologist-authored reports. These findings align with a growing body of literature suggesting that Large Language Models (LLMs) can effectively bridge the health literacy gap in radiology [14,27]. By using a prompt derived from real-world patient questions (“Can you explain this radiology report in simple terms?”), the intervention mirrored the actual information-seeking behavior of patients [21]. This confirms that the technical barrier of “medicalese” can be successfully dismantled by current generation AI tools, validating the feasibility of patient-centered reporting initiatives [28,29,30,31].

A key innovation of this study is that it evaluates AI-based radiology report simplification using a randomized trial design rather than relying only on readability scores, expert judgment, or patient preference. The study also focuses on a brain MRI/MRV report, a clinically complex report type that may contain terminology capable of creating uncertainty for patients. In addition, this trial extends prior work by examining not only whether AI simplification improves understanding, but also whether it changes anxiety after report exposure. By measuring general health literacy, radiology literacy, report understanding, and anxiety together, the study provides a more complete assessment of how patients process AI-simplified radiology information. This combined focus on comprehension, psychological response, and literacy-related factors represents an important contribution to patient-centered radiology communication.

### 4.2. Refuting the “Confusion Causes Anxiety” Hypothesis

However, the most profound finding of this study concerns the relationship between understanding and anxiety. For decades, the radiology community has hesitated to provide direct patient access to reports, citing the concern that the complexity of medical terminology would cause unnecessary distress [32]. A recent systematic review highlights that this paternalistic caution has been a dominant theme in radiology for over 20 years, with physicians consistently arguing that patients need a “filter” to prevent panic caused by misunderstanding [20]. The prevailing hypothesis has been that anxiety is a product of confusion that patients are scared because they do not know what the words mean [32].

Our results challenge this hypothesis. We observed a statistically significant positive correlation between report understanding and anxiety (r = 0.182, *p* < 0.001). This suggests that as patients understood the report better, their anxiety increased rather than decreased. Even more striking was the relationship with health literacy: participants with higher general health literacy, those best equipped to navigate medical information, reported significantly higher anxiety levels (r = 0.283, *p* < 0.001). If confusion were the primary driver of distress, the AI-simplified group or the high-literacy cohort should have been the calmest. Instead, we found that clarity can heighten vigilance.

### 4.3. The Paradox of Clarity

This phenomenon, which we term the “paradox of clarity,” challenges the foundational assumption of the 21st Century Cures Act that transparency is inherently empowering. Our data suggest that for many patients, the “black box” of medical terminology may actually serve a protective function. The “uncertainty management theory” posits that in some health contexts, uncertainty can buffer anxiety by allowing patients to maintain optimistic biases [16]. When a report is simplified, terms like “mass,” “lesion,” or “signal abnormality” are stripped of their ambiguity. While the patient now intellectually understands the definition of the finding, they often lack the clinical experience to understand its significance (e.g., that a “T2 hyperintensity” might be a harmless incidental finding) [21].

This validates the historical concerns of referring physicians, who have long argued that report access without clinical context leads to “scanxiety” [15]. Previous studies have shown that patients often turn to the internet to interpret their results, a behavior that frequently exacerbates distress [33]. Our study adds a critical nuance: even when the “internet search” is replaced by a high-quality AI simplification, the anxiety remains. This indicates that the problem is not misinformation (which AI solves), but information itself when severed from reassurance. The patient who understands “I have a lesion” but does not know “It is benign” is in a worse psychological state than the patient who simply waits for the doctor.

### 4.4. The Burden of Knowledge: Health Literacy and Anxiety

The strong correlation between health literacy and anxiety further complicates the narrative. Participants with higher health literacy scores who are arguably the “ideal” candidates for patient portals experienced the most distress. This may be because highly literate patients are more aware of the potential implications of medical findings [32]. They are “monitors” rather than “blunters” in their coping style actively scanning for threats [16]. In the context of a radiology report, this vigilance backfires. A simplified report gives them just enough information to construct a frightening scenario, but not enough clinical data to deconstruct it. This finding aligns with research in oncology, where highly educated patients often report higher distress because they fully grasp the gravity of prognostic statistics [33].

### 4.5. Re-Evaluating the Role of the Radiologist

These findings suggest that the radiology community must reframe its approach to patient-centered care. If AI solves the literacy problem but exacerbates the emotional problem, the radiologist’s value proposition shifts. Radiologists are no longer needed solely as translators of jargon; AI can perform that task faster and, arguably, more consistently. Instead, the radiologist is needed as a “contextualizer” [34].

The “direct communication” model, where radiologists speak with patients, has often been dismissed as impractical due to workflow constraints [34]. However, our results suggest that without some form of clinical guidance, the “simplified report” is an incomplete intervention. As noted in prior literature, patients do not just want to know what is in the image; they want to know what it means for their life [35]. An AI can define “stenosis,” but only a clinician can say, “You don’t need to worry about that.” Therefore, we propose that AI-simplified reports should not be viewed as a standalone replacement for physician interaction. Instead, they are best utilized as a preparatory tool. A patient who reads a simplified report is better equipped to ask informed questions during a follow-up visit. The goal of simplification should be to shift the doctor–patient conversation from “What does this word mean?” to “What do we do next?” [36].

### 4.6. Ethical and Privacy Considerations of Chatbot-Based Simplification

Ethical and privacy considerations are central to the use of chatbot-based radiology report simplification. In this study, the report was fully de-identified before AI simplification, and no identifiable health information was entered into the AI system. However, real-world clinical use would require stronger safeguards, including secure institutional platforms, clear privacy policies, avoidance of entering identifiable patient information into non-approved public chatbot systems, and clinician oversight. AI-generated summaries should also be reviewed carefully because simplification errors, omitted details, or overly reassuring wording could affect patient understanding and safety. Therefore, chatbot-based report simplification should be implemented as a governed communication-support tool rather than an unmonitored replacement for professional interpretation.

### 4.7. Limitations and Future Directions

Several limitations should be acknowledged. First, this study was conducted using a hypothetical online scenario rather than real clinical patients receiving their own imaging results. Therefore, although anxiety was assessed using items adapted from recognized anxiety- and distress-related measures, participants’ emotional responses may not fully capture the intensity of real-world scanxiety, particularly when abnormal findings are linked to personal health consequences. Second, the study used a single de-identified brain MRI/MRV report, which may limit generalizability to other imaging modalities, report types, anatomical regions, and disease contexts. Third, the intervention was generated using one AI model, ChatGPT, and the findings may not apply equally to other large language models or AI-based simplification tools. Fourth, the online participant sample may not fully represent patients with different levels of health literacy, digital access, cultural backgrounds, or clinical vulnerability.

Despite these limitations, the finding that anxiety was positively associated with understanding, even when measured using recognized anxiety-related items in a low-stakes hypothetical setting, suggests that the emotional impact of clearer radiology information deserves further study in real clinical environments. Future research should test AI-simplified reports among actual patients receiving genuine imaging results, include multiple report types and clinical scenarios, compare different large language models, and incorporate qualitative methods to better understand patients’ emotional experiences. Future studies should also evaluate hybrid communication models in which AI-generated plain-language summaries are paired with clinician-reviewed context, reassurance cues, clear next-step recommendations, or direct links to physician follow-up. Longer follow-up periods may also help determine whether AI simplification has delayed psychological effects beyond the immediate post-report response.

## 5. Conclusions

In conclusion, this randomized trial showed that AI-based simplification of a brain MRI report significantly improved participant understanding compared with the original radiology report. However, this improved comprehension did not translate into lower anxiety, as anxiety scores were similar between the original report and AI-simplified report groups. This finding suggests that simplifying medical terminology alone may not be sufficient to reduce the emotional burden associated with reading radiology results.

These results highlight an important distinction between understanding and reassurance. AI can help patients understand what a radiology report says, but it may not fully explain what the findings mean for the patient’s prognosis, urgency, or next steps. Therefore, AI-simplified reports should be viewed as supportive communication tools rather than replacements for clinician guidance. Patient-centered radiology communication requires not only clearer language, but also appropriate clinical context, follow-up, and reassurance from healthcare professionals.

## Figures and Tables

**Table 1:** Patient-like radiology-report clarification questions with ChatGPT and Gemini rankings (1 = most frequent). The top-ranked question was used as the standard simplification prompt.

Questions	ChatGPT	Gemini
Can you explain this radiology report in simple terms?	1	1
Is this report normal or are there any concerning findings?	2	5
What does this finding mean? (often followed by specific terms)	3	6
Is there anything I should be worried about in this radiology report?	4	7
What does [specific term] mean in my report?	5	3
Can you help me understand the impression/conclusion section of this report?	6	2
Can you summarize the main findings of this report?	7	4
What is the significance of the findings in this report?	8	8

**Table 2:** Sociodemographic characteristics of participants.

Characteristic	Category	*n*	% (Valid)
Age (years) (*n* = 803)	<20	5	0.6
	20–29	28	3.5
	30–39	490	61.0
	40–49	255	31.8
	50–59	13	1.6
	60–69	5	0.6
Gender (*n* = 803)	Male	599	74.6
	Female	204	25.4
Race (*n* = 803)	White	780	97.1
	Black or African American	9	1.1
	American Indian or Alaska Native	3	0.4
	Asian	4	0.5
	Native Hawaiian or Pacific Islander	2	0.2
	Unknown	3	0.4
	Other	2	0.2
Highest education (*n* = 803)	High school or below	65	8.1
	Bachelor’s degree	557	69.4
	Master’s or PhD	98	12.2
	Any health-related degree	83	10.3
Annual household income (*n* = 803)	<$10,000	48	6.0
	$10,000–24,999	61	7.6
	$25,000–49,999	154	19.2
	$50,000–74,999	181	22.5
	$75,000–99,999	277	34.5
	≥$100,000	82	10.2
Language spoken at home (*n* = 803)	English	655	81.6
	Other	148	18.4
Religious preference (*n* = 803)	Formal religious group	413	51.4
	No formal religion	131	16.3
	Spiritual but not religious	188	23.4
	Data not available/NA	71	8.8

**Table 3:** Main outcomes by randomized group. Report understanding (0 to 7), Health Literacy (0–36), Anxiety (1–5).

Outcome	Control Group (*n* = 402) Mean ± SD	AI-Modified Report (*n* = 401) Mean ± SD	*p*-Value
Report understanding	5.61 ± 1.49	5.78 ± 1.31	0.007
Radiology literacy	9.03 ± 2.07	9.13 ± 2.02	0.276
Health literacy	20.68 ± 3.63	20.86 ± 3.77	0.820
Anxiety	3.23 ± 0.85	3.24 ± 0.84	0.103

**Table 4:** Health literacy level.

Health Literacy Level	Control, N (%)	AI Modified, N (%)	Total, N (%)
Inadequate	36 (9%)	39 (9.7%)	75 (9.3%)
Marginal	256 (63.7%)	253 (63.1%)	509 (63.4%)
Adequate	110 (27.4%)	109 (27.2%)	219 (27.3%)

## Data Availability

The data that support the findings of this study are available from the corresponding author upon reasonable request.

## Supplementary Materials

The following supporting information can be downloaded at: https://www.mdpi.com/article/10.3390/jcm15114158/s1. The survey used for this study.
