# Supplementary material for: Psychological Impact of AI-Simplified Brain MRI Reports: A Randomized Trial of Patient Understanding, Anxiety, and Health Literacy
Source: J Clin Med. 2026 May 28;15(11):4158. doi: 10.3390/jcm15114158 (PMC13258259; doi:10.3390/jcm15114158)
Supplement: Supplementary file 1 [file jcm-15-04158-s001.zip › jcm-4296036-supplementary.pdf]

Supplementary Material

Start of Block: Sociodemographic for all groups

Age

- <20 years
- 20–29 years
- 30–39 years
- 40–49 years
- 50–59 years
- 60–69 years
- 70+ years

Gender

- Male
- Female

Race

- White
- Black or African American
- American Indian or Alaska Native
- Asian
- Native Hawaiian or Pacific Islander
- Unknown
- Other

What best describes your religious preferences? (check one)

- I belong to a formal religious group
- I do not have a formal religion, nor am I a spiritual person
- I consider myself spiritual, but not religious
- Data not available

Is English the language you most commonly speak at home?

- Yes
- No

Income

- ◯ Less than $10,000
- ◯ $10,000–$24,999
- ◯ $25,000–$49,999
- ◯ $50,000–$74,999
- ◯ $75,000–$99,999
- ◯ $100,000 or more

What is your highest level of education?

- ◯ High school or below
- ◯ Bachelor’s degree (not related to the health field)
- ◯ Master’s or PhD (not related to the health field)
- ◯ Any degree in a health-related field

End of Block: Sociodemographic for all groups

Start of Block: Control Group

Gender: Male
Date of Birth: XXXXX

Procedure: MR0020, MRI BRAIN; MRI ANGIO BRAIN MRV; MRI ANGIO BRAIN CIRCLE OF W ILLIS
Medical Question: slurred speach, 6th nerve palsy and balance problem and weaakness of both Lower limbs
Clinical Comment: ; ...

=========== REPORT TEXT ===========
MRI BRAIN of 01-JAN-2024:
MRI ANGIO BRAIN COW of 01-JAN-2024:

MRI ANGIO BRAIN MRV of 01-JAN-2024:

CLINICAL INDICATION:
Slurred speech, 6th nerve palsy, balance problem and weakness of lower limbs.
COPMARISION: None.
TECHNEQE:
Brain MRI, MRV and MRA performed pre and post contrast.

FINDINGS:
There is a large dilated vascular structure extending from the left choroid fissure along the left ambient cistern into the left CP angle with aneurysmal dilatation seen along the left dorsal aspect of the pons, measuring about 1.5 x 1.3 cm on the axial plane. Severe mass effect is seen on the left aspect of the midbrain and pons with associated deformity of the dorsal pons and focal brainstem edema. There is also mass effect on the lower aspect of the cerebral aqueduct but with no evidence of supratentorial hydrocephalous.

This dilated vascular structure demonstrates flow signal intensity on time-of-flight MRA with evidence of arterialization on SWI, denoting presence of arteriovenous shunting with imaging features highly suggestive of arteriovenous fistula. There is also flow signal intensity noted on

time-of-flight MRA along the left superior petrosal sinus extending into the left sigmoid sinus with prominent veins along the floor of the left middle cranial fossa. There is also flow signal intensity noted along the left basal of Rosenthal and vein of Galen. Increased vascularity is also noted at the left thalamus on the postcontrast images with a microhemorrhage noted along its ventral aspect.

The main arterial feeder is probably the left anterior choroidal artery with possible contribution from the left PCA. The left middle meningeal artery is also enlarged and likely contributing to the fistulous communication.

There are periventricular and subcortical white matter patchy high T2/FLAIR signal intensity, likely related to small vessel disease. No acute diffusion abnormality.

IMPRESSION:
Findings highly suggestive of a complex arteriovenous fistula with aneurysmal dilatation, resulting in severe mass effect and deformity of the brain stem with associated focal brainstem edema. Neuro-intervention consultation and further evaluation by catheter cerebral angiogram is recommended.

End of Block: Control Group

Start of Block: Standard ChatGPT

1. **Reason for the Scan**: The scan was done because the patient had slurred speech, problems with balance, weakness in the lower limbs, and a condition affecting the 6th cranial nerve (which controls eye movement).
2. **Findings**:
    - There's a large, abnormal blood vessel on the left side of the brain, near a structure called the *pons* (part of the brainstem). It has a ballooned (aneurysmal) area about 1.5 cm in size. This vessel is putting pressure on nearby brain structures, causing slight swelling in the brainstem.
    - This blood vessel issue is likely an *arteriovenous fistula* (an abnormal connection between an artery and a vein) that shows signs of shunting, meaning blood is flowing in an unusual pattern between the artery and vein.
    - Other blood vessels nearby are also enlarged, indicating higher-than-normal blood flow in the area.
    - There's some increased vascularity (blood supply) near a part of the brain called the *thalamus*, with a small spot of bleeding.
    - The white matter in the brain (brain tissue) shows some small patches that may be related to small blood vessel disease, common in older adults, but there are no signs of a recent stroke.
3. **Conclusion**:
    - The findings strongly suggest a complex arteriovenous fistula with an aneurysm, which is affecting the brainstem area. This condition may require further tests or treatment, and it's recommended that the patient consult a specialist in neuro-intervention (a doctor who treats blood vessel issues in the brain).

This type of issue can cause symptoms like the ones the patient is experiencing due to the pressure and altered blood flow in the brain. A more detailed test called a catheter angiogram is suggested to confirm these findings and plan treatment.

End of Block: Standard ChatGPT

Start of Block: Quiz

The next short, ungraded quiz is based on the report you just viewed. Feel free to go back to it to find the answers

1. What is an arteriovenous fistula (AVF)?

- ○ A. A normal pathway for blood in the brain
- ○ B. An abnormal connection that lets blood go directly from an artery to a vein
- ○ C. A broken nerve in the brainstem
- ○ D. A blood clot blocking a vein

---

2. Which brain area is being pressed by the abnormal vessels?

- ○ A. The brainstem
- ○ B. The spinal cord
- ○ C. The right cerebellum
- ○ D. The skull bones

---

3. On which side is the problem mainly described?

- ○ A. Right side
- ○ B. Left side
- ○ C. Both sides equally
- ○ D. Side not mentioned

---

4. Which symptom best fits pressure on the brainstem in this case?

- A. Better memory
- B. Improved arm strength
- C. Problems with balance and eye movement
- D. Tingling that comes and goes in the fingers only

---

5. What did the scan say about a new (“fresh”) stroke?

- A. A new stroke is clearly present
- B. A new stroke cannot be ruled out
- C. No evidence of a new stroke was seen
- D. The scan did not look for stroke

---

6. Which imaging was used to look at the brain and blood vessels?

- A. X-ray only
- B. CT scan only
- C. MRI with special blood-vessel pictures
- D. Ultrasound of the neck only

---

7. What is the recommended next step to evaluate the vessel problem more closely?

- ○ A. Start surgery immediately
- ○ B. Catheter cerebral angiogram (a dye test of the brain vessels)
- ○ C. Repeat plain X-ray next week
- ○ D. No further tests are needed

End of Block: Quiz

Start of Block: Anexity

Please think about how the information in this radiology report might affect its reader (the patient whose report it is). Indicate to what extent you believe the following feelings could occur.

To what extent might the information in this report cause the reader to feel worried about their health?

- ○ Not at all
- ○ A little
- ○ Moderately
- ○ Quite a lot
- ○ Extremely

To what extent might the information in this report make the reader feel restless or uneasy?

- ◯ Not at all
- ◯ A little
- ◯ Moderately
- ◯ Quite a lot
- ◯ Extremely

To what extent might reading this report lead the reader to think more about their pain than usual?

- ◯ Not at all
- ◯ A little
- ◯ Moderately
- ◯ Quite a lot
- ◯ Extremely

End of Block: Anexity

Start of Block: Health Literacy

Next is a short set of multiple choice questions to gauge your general health literacy

---

How often are appointment slips written in a way that is easy to read and understand?

- ◯ Always
- ◯ Often
- ◯ Sometimes
- ◯ Occasionally
- ◯ Never

How often are medical forms difficult to understand and fill out?

- ◯ Always
- ◯ Often
- ◯ Sometimes
- ◯ Occasionally
- ◯ Never

How often do you have difficulty understanding written information your health care provider gives you?

- ◯ Always
- ◯ Often
- ◯ Sometimes
- ◯ Occasionally
- ◯ Never

How often do you have problems learning about your medical condition because of difficulty understanding written information?

- Always
- Often
- Sometimes
- Occasionally
- Never

How confident are you filling out medical forms by yourself?

- Always
- Often
- Sometimes
- Occasionally
- Never

How confident do you feel you are able to follow the instructions on the label of a medication bottle?

- Always
- Often
- Sometimes
- Occasionally
- Never

How often do you have someone help you read hospital materials?

- ○ Always
- ○ Often
- ○ Sometimes
- ○ Occasionally
- ○ Never

End of Block: Health Literacy

Start of Block: literacy on radiology reports

The following questions will show how far your knowledge in the radiology field

Radiological modality that uses a magnetic field to create images of the inside of your body is

- ○ CT
- ○ MRI
- ○ Nuclear Medicine
- ○ don’t know

Radiological modality that uses X-ray to create images of the inside of your body is

- ○ CT
- ○ MRI
- ○ Nuclear Medicine
- ○ don’t know

Radiological modality that uses small amounts of radioactive material to create images of the inside of your body is

- ○ CT
- ○ MRI
- ○ Nuclear Medicine
- ○ don’t know

All radiology modalities use radiation in the scans

- ○ Yes
- ○ No
- ○ don’t know

There is no limit to do many X-ray scans in per year

- ◯ Yes
- ◯ No
- ◯ don’t know

The body can filtrate all the radiation from the body at the end of the imaging scan day

- ◯ Yes
- ◯ No
- ◯ don’t know

Please, answer the following two questions based on this image

---

This is a kidney image

- ◯ Yes
- ◯ No
- ◯ don’t know

It's normal that radiology images appear in white and gray

- ◯ Yes
- ◯ No
- ◯ Don't know

All the imaging tests have the same preparation instructions

- ◯ Yes
- ◯ No
- ◯ Don't know

The radiology scan that requires no metal on the body is

- ◯ Ultrasound
- ◯ MRI
- ◯ Don't know

End of Block: literacy on radiology reports

Start of Block: Random ID

Here is your ID: ${e://Field/Random%20ID}
Copy the value to paste on MTurk
When you have copied this ID, please click the next button to submit your survey

End of Block: Random ID

Start of Block: test id

Here is your ID: ${e://Field/Random%20ID}
Copy the value to paste on MTurk
When you have copied this ID, please click the next button to submit your survey

End of Block: test id
